# Trends in low-energy mechanisms of injury at the regional trauma center in Stockholm, Sweden during 2014–2023

**DOI:** 10.1007/s00068-025-03067-3

**Published:** 2026-01-29

**Authors:** Sara Bredberg, Denise Nilsson, Aurora Sandart, Filip Christiansen, Denise Bäckström, Rebecka Rubenson Wahlin, Oscar Lapidus

**Affiliations:** 1 Department of Clinical Science, Intervention and Technology, Karolinska Institutet, Stockholm, Sweden; 2 Department of Biomedical and Clinical Sciences, Linköping University, Linköping, Sweden

**Keywords:** Geriatric trauma, Low-energy, Mechanism of injury, Regional trauma center

## Abstract

**Background:**

The Swedish population is aging, and elderly individuals are more likely to sustain injuries from low-energy mechanisms of injury. Trauma has traditionally been a disease of the young, but elderly patients now account for the majority of deaths from external causes in Sweden. No recent studies have examined whether this demographic shift has translated into a higher rate of low-energy mechanisms of injury in the trauma population.

**Aim:**

To investigate temporal trends in the proportion of low-energy mechanisms of injury at the regional trauma center in Stockholm during 2014–2023.

**Methods:**

13188 patients treated at Karolinska University Hospital in Solna were obtained from the Swedish Trauma Registry. The annual proportion of patients with a low-energy mechanism of injury was calculated, and the temporal trends were assessed using weighted linear regression models. Two subgroups were also defined to separately analyze primary transports and patients with a blunt mechanism of injury.

**Results:**

The overall proportion of low-energy mechanisms in the cohort was 15% (*n* = 1978). No statistically significant temporal trends were observed in the study cohort (R^2^ = 0.07, *p* = 0.477), or in any of the examined subgroups of primary transport (R^2^ = 0.35, *p* = 0.073) or patients with blunt mechanism of injury (R^2^ = 0.29, *p* = 0.108).

**Conclusion:**

There were no significant temporal trends in the rate of low-energy mechanisms of injury in patients treated at the regional trauma center during 2014–2023. Whether these findings are representative of the wider regional trauma cohort is uncertain.

## Background

Life expectancy in Sweden is increasing, with a steadily aging Swedish population [1]. According to Statistics Sweden, the proportion of citizens aged ≥ 70 years is expected to increase from the current 15% to 20% by 2070 [2]. Previous studies indicate this demographic transition may also be reflected in trauma populations worldwide, with evidence of aging trauma cohorts across several healthcare systems globally [3–7]. Trauma has traditionally been considered to be a disease of the young, but elderly patients now account for the majority of deaths from external causes in Sweden [8]. For example, in 2023 approximately 60% of those who died from external causes were at or above 65 years of age, and approximately 40% were 80 years or older [8].

Previous investigations suggest geriatric trauma patients differ substantially from traditional trauma populations, and that low-energy falls are a common cause of serious injuries in the elderly [3, 9, 10]. One study reported ground-level falls to account for 50% of traumatic deaths among geriatric patients and addition to being the most prevalent mechanism of injury in this group [9]. However, there is limited evidence regarding the role of modern trauma care in the management of patients with severe injuries sustained from low-energy mechanisms of injury [11, 12]. This is an increasingly relevant concern for healthcare providers, but no recent studies have examined whether the demographic shift in Sweden has translated into a higher rate of low-energy mechanisms of injury in the trauma population.

## Aim

This study aimed to investigate temporal trends in the proportion of low-energy mechanisms of injury at the regional trauma center in Stockholm during 2014–2023.

## Methods

### Study design

The present study was performed as a single-center retrospective cohort study analyzing data from the Swedish Trauma Registry (SweTrau) [13]. The study was reported in accordance with the Strengthening the Reporting of Observational studies in Epidemiology (STROBE) guidelines [14].

### Ethical considerations

Prior to initiation, this study was approved by Swedish Ethical Review Authority in Sweden (2022–06727-01, 2024–02482-01).

### Setting

The present study examined data from the regional trauma center in Stockholm, Sweden, with a population of 2.5 million residents in the metropolitan area [15]. Trauma care in Stockholm is centralized to Karolinska University Hospital in Solna, the regional trauma center, in accordance with a prehospital transport directive used by emergency medical services (EMS) in the Stockholm region [16]. The prehospital transport directive has been described by previous authors and has remained largely unchanged since described in a previous study [17]. In addition to the regional trauma center, Stockholm has four non-trauma-center hospitals close to the urban core, as well as two additional hospitals located in Norrtälje and Södertälje in the geographic edges of the Stockholm metropolitan area.

### Participants

The study cohort included all registered trauma patients ≥ 15 years old treated at the regional trauma center hospital in Stockholm (Karolinska University Hospital in Solna) during 2014–2023. Patients with an unknown mechanism of injury were excluded. Two subgroups were defined and analyzed separately; the first subgroup comprised only severely injured patients, defined as a New Injury Severity Score (NISS) > 15, who were primarily transported to the regional trauma center (no secondary transports). The second subgroup also constituted primary transports with NISS > 15 but only included patients with blunt injuries (i.e. no penetrating injuries).

### Variables

The primary outcome was change in the proportion of low-energy mechanisms of injury over time; low-energy mechanisms were defined as the primary mechanism of injury being a low fall according to the ‘inj_mechanism’ variable in the registry. SweTrau defines low falls as a fall from less than 1.5 × patient height which also includes ground-level falls [18]. The proportion of low-energy mechanism of injury each year was then calculated by dividing the number of patients with low-energy mechanisms of injury by the total number of patients in the cohort or subgroup. Primary transports were identified using the ‘pre_transport’, ‘hosp_transferred’ and ‘TraumaAlarmCriteria’ variables in the SweTrau registry. The technical details related to the coding of these variables can be found in the SweTrau registry manual [18]. Injury severity was defined as NISS > 15 using the coded value of the ‘NISS’ variable. Blunt and penetrating injuries were differentiated using ‘inj_dominant’.

### Data sources

SweTrau is a national quality registry which aims to monitor the quality of trauma care in Sweden by collecting and providing patient data for research purposes [13]. The registry includes all patients who prompt an in-hospital trauma team activation, and all patients with NISS > 15 regardless of trauma team activation status, with some exclusion criteria as described on the website and in the registry manual [13, 18].

### Bias

Restricting the analysis to only include patients treated at the regional trauma center may have introduced some degree of selection bias resulting from prehospital triage tools (i.e. the prehospital transport directive for trauma patients in Stockholm). Studies have shown that geriatric trauma patients are at risk of undertriage [19–23]. This could result in disproportionate selection against geriatric trauma, leading to systematic underrepresentation of elderly patients at the regional trauma center. This would reasonably affect the results of the present study and this issue is further expanded upon in the discussion section.

### Study size

In the present study, all patients registered in SweTrau during 2014–2023 were assessed for eligibility to be included in the study. Patients registered prior to 2014 were not included in the analysis due to uncertainty regarding the quality of data. Including patients treated after 2023 was deemed beyond the scope of the present investigation as this was not readily available to the authors at the time of the study.

### Statistics

Descriptive statistics were calculated and presented across background variables using appropriate measures of central tendency and dispersion (i.e. frequencies, medians and quartiles). The Shapiro-Wilk test was used to assess for Gaussian distribution. Temporal trends in mechanisms of injury were assessed using linear regression weighted to the number of patients each year; the study year (i.e. 2014 recoded as 0) was used as the independent variable in the regression model and the proportion of low-energy falls each year was used as the dependent variable. Using the relative study year instead of calendar year was done to center the regression equation and provides a more intuitive understanding of the regression constant. Data management and statistical analysis was performed using the Python programming language and IBM SPSS v29. A significance level of 0.05 was used for all statistical analyses. Figures were created using DataGraph.

## Results

A total of 13,188 patients were included in the study, of which 69% (*n* = 9126) were male and 31% (*n* = 4062) were female patients. The median age was 42 years, with 20% (*n* = 2649) aged ≥ 65 years and 7.1% (*n* = 934) aged ≥ 80 years (Table 1). Baseline characteristics of the study cohort and the two subgroups are presented in Table 1. There was no statistically significant increase in age during the study period (0.097).

The overall proportion of low-energy mechanisms of injury in the study cohort was 15% (*n* = 1978). In the examined subgroups, the corresponding proportions were 13% (*n* = 492) among primary transports with NISS > 15 and 16% (*n* = 491) in the NISS > 15 blunt mechanism group (Table 1). Low falls was the third most common mechanism of injury in the overall study cohort and the second most common in both subgroups. Low falls were particularly prevalent among older patients, accounting for 45% of cases in patients ≥ 65 years old and 60% of cases in patients aged ≥ 80 years.

**Fig. 1:**
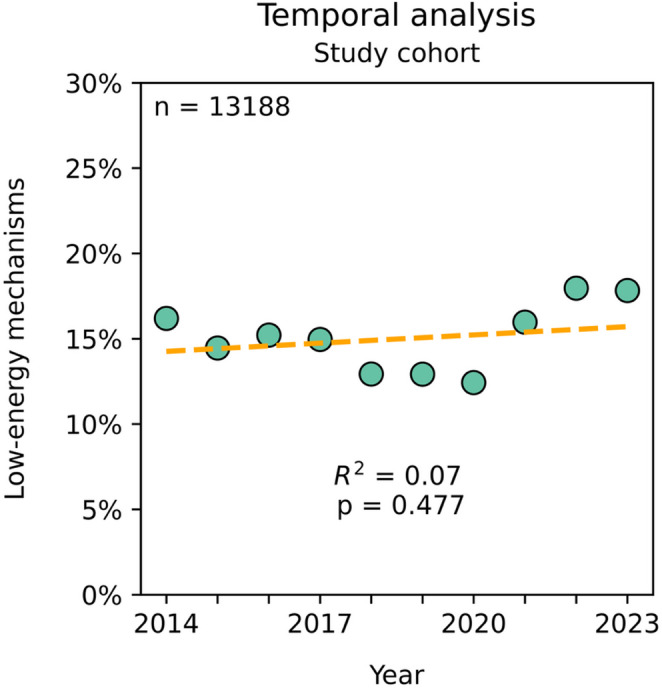
Temporal trend in the proportion of low-energy mechanisms of injury during 2014–2023 in the study cohort, with weighted regression line

**Fig. 2:**
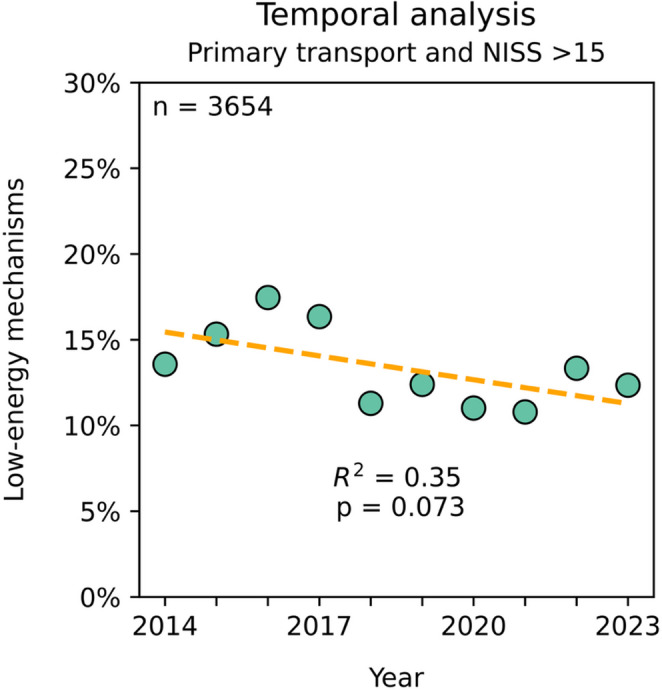
Temporal trend in the proportion of low-energy mechanisms of injury during 2014–2023 in the subgroup of primary transport and NISS > 15 patients, with weighted regression line

**Fig. 3:**
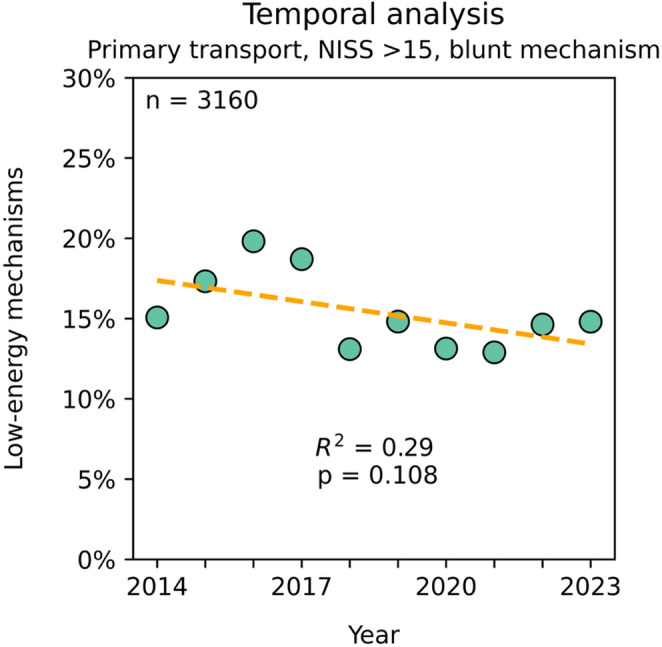
Temporal trend in the proportion of low-energy mechanisms of injury during 2014–2023 in the subgroup of patients with blunt mechanism of injury and NISS > 15, with weighted regression line

There was no statistically significant change in the rate of low-energy mechanism of injury during 2014–2023 in the study cohort (R^2^ = 0.07, *p* = 0.477) (Fig. 1; Table 3). Similarly, no significant trends were observed in the primary transport subgroup (R^2^ = 0.35, *p* = 0.073) or in the blunt subgroup (R^2^ = 0.29, *p* = 0.108) (Figs. 2 and 3).

## Discussion

This study aimed to investigate temporal trends in the proportion of low-energy mechanisms of injury at the regional trauma center in Stockholm during 2014–2023. There were no statistically significant trends in the rate of low-energy mechanisms of injury in the overall study cohort or in any of the examined subgroups.

While this topic has not previously been explicitly examined in a Swedish context and consequently limiting the availability of comparable data, one recent report from the Australian Institute of Health and Welfare found that the incidence of hospitalization after injuries sustained from falls (of which almost 70% were considered low-energy mechanisms of injury) increased by 2–3% per year [24]. Considering the similarities between Australia and Sweden (i.e. developed countries, aging populations), there should arguably also be an increase in low-energy trauma in Sweden [1, 24, 25]. However, the present study found no evidence of an increasing proportion of low-energy mechanisms of injury in patients treated at the regional trauma center. This finding was unexpected in the context of the steadily aging population in Sweden [1].

In the present study, the overall proportion of low-energy mechanisms of injury in the study cohort was 15%, and 13–16% in the examined subgroups. Low falls was the third most common mechanism of injury overall, accounting for 15% of total cases. For contrast, a previous study of trauma patients at the largest non-trauma-center hospital in Stockholm found that low falls accounted for 56% of cases in the severely injured trauma cohort during 2019–2022, with a median patient age of 71 years [23]. This finding is markedly higher than the 13–16% observed in the present study, highlighting the differences in patient demographics between the regional trauma center and various non-trauma-center hospitals in the Stockholm trauma system. The demographic difference between these two hospitals operating in the same area appears indicative of a trauma system which disproportionately triages geriatric trauma patients to non-trauma-center hospitals rather than the trauma center. Consequently, this raises concerns about whether data from a single trauma center in an organized regional trauma system is representative of the broader Swedish trauma population, given that prehospital triage practices seem to systematically select against direct trauma center transport of elderly trauma patients.

As previously stated, this study found no significant change in the rate of low-energy mechanisms of injury during 2014–2023 at the regional trauma center in Stockholm. However, the present study examined a single trauma center cohort in an organized regional trauma system with strict prehospital selection of major trauma patients in accordance with the transport directive used by EMS in the Stockholm region [16]. This transport directive states that all severely injured patients in Stockholm should be transported to the regional trauma center, instead of to the nearest hospital [16]. This selection is based on pre-defined physiological and anatomical criteria explicitly stated in the trauma transport directive, with emphasis on compromised vital signs and high-energy mechanisms of injury. Consequently, a possible explanation for why the rate of low-energy mechanisms found to not increase over time at the regional trauma center may be that these patients are simply transported somewhere else (i.e. non-trauma-center hospitals). This interpretation is consistent with findings from one study in the United States where geriatric patients were found to be at increased risk of prehospital undertriage, resulting in fewer patients being triaged to trauma center transport in the early phase of trauma management [22]. To fully assess whether low-energy trauma is increasing in Sweden, system-wide analyses across all hospitals in a regional trauma system, rather than trauma center cohorts alone, are needed to minimize prehospital selection bias. This was deemed beyond the scope of the present investigation due to limited longitudinal data from other hospitals in the Stockholm trauma system; however, this warrants further investigation.

The accuracy of prehospital triage in Stockholm has previously been evaluated [17]. However, the ability of the current triage system to identify severely injured geriatric patients remains uncertain. The question of whether these patients benefit from trauma center care is also debatable. In general, there is limited evidence regarding which patients benefit from trauma centers; in the context of low-energy trauma specifically, the evidence base is even more limited, as concluded by one recent systematic review investigating the potential survival benefit of different levels of trauma centers in elderly patients after low-level falls [11]. In addition, another study by Mehta et al. found no survival difference between patients treated for low falls at level 1 and level 2 trauma centers [12]. On the other hand, as trauma centers have unequivocally been shown to improve survival for trauma patients across a wide spectrum of injuries with the greatest benefit for the most severely injured patients, one may argue that injury severity, regardless of mechanism of injury or energy level, should be the primary determinant for transport to the regional trauma center [26].

In the context of a rapidly aging population, future studies are of great importance to determine whether elderly patients with low-energy trauma experience improved outcomes when treated at trauma centers compared. This is of importance not only to ensure these vulnerable patients receive the most appropriate care, but also to allow for optimal evidence-based allocation of limited healthcare resources.

## Conclusion

There were no significant temporal trends in the rate of low-energy mechanisms of injury in patients treated at the regional trauma center during 2014–2023. Whether these findings are representative of the wider regional trauma cohort is uncertain due to prehospital selection bias and warrants further investigation.

## Data Availability

The data will not be published but is available from the corresponding author.

## Appendix

**Table 1:** Baseline characteristics of the trauma cohort at the regional trauma center in Stockholm during 2014–2023

Demographics	Study cohort *n* = 13,188		Primary transport and NISS > 15 *n* = 3654		Primary transport, blunt mechanism and NISS > 15 *n* = 3160	
Patient characteristics:						
Sex (male: female), % (n)	69:31	(9126:4062)	75:25	(2756:898)	73:27	(2296:864)
Age, median (Q1, Q3)	42	(27, 60)	48	(29, 65)	51	(33, 68)
Age ≥ 65, % (n)	20%	(2649)	26%	(955)	29%	(931)
Age ≥ 80, % (n)	7.1%	(934)	9.3%	(340)	11%	(339)
Injury severity:						
NISS, median (Q1, Q3)	9	(3, 22)	27	(19, 34)	27	(19, 34)
NISS > 15, % (n)	36%	(4779)	100%	(3654)	100%	(3160)
Mechanism of injury:						
Low-energy, % (n)	15%	(1978)	13%	(492)	16%	(491)
Blunt injury, % (n)	86%	(11281)	86%	(3160)	100%	(3160)

**Table 2:** Mechanism of injury for patients in the study cohort and subgroups

	Study cohort *n* = 13,188		Primary transport and NISS > 15 *n* = 3654		Primary transport, blunt mechanism and NISS > 15 *n* = 3160	
Mechanism of injury, % (*n*):						
High fall	22%	(2905)	28%	(1038)	33%	(1037)
Motor vehicle accident	17%	(2232)	9.9%	(363)	11%	(363)
Low fall	15%	(1978)	14%	(492)	16%	(491)
Injured by knife/sharp object	11%	(1445)	8.0%	(294)	0.1%	(2)
Hit/struck by blunt object	9.7%	(1278)	9.0%	(330)	10%	(330)
Bicycle accident	7.9%	(1041)	8.4%	(307)	9.7%	(307)
Motorcycle accident	7.7%	(1010)	9.4%	(345)	11%	(345)
Injured pedestrian	3.6%	(476)	4.7%	(170)	5.4%	(170)
Gun shot wound	3.4%	(453)	5.4%	(196)	0%	(0)
Explosion injury	0.3%	(44)	0.3%	(12)	0.3%	(9)
Other vehicle	1.1%	(143)	1.2%	(44)	1.4%	(44)
Other mechanism of injury	1.4%	(183)	1.7%	(63)	2.0%	(62)

**Table 3:** Numeric results from weighted linear regression analyses of change in proportion of low-energy mechanisms of injury during 2014–2023. Note that the calendar year (2014–2023) has been centralized (0–9)

Group	Constant	Coefficient (95% CI)		*p*-value	*R* ^2^
Study cohort	14.32	0.16	(−0.34–0.66)	0.477	0.07
Primary transport and NISS > 15	15.48	−0.46	(−0.98–0.05)	0.073	0.35
Blunt mechanism and NISS > 15	17.44	−0.44	(−1.00–0.12)	0.108	0.29

## Abbreviations

EMS: Emergency medical services
NISS: New Injury Severity Score
SweTrau: The Swedish Trauma Registry
TTA: Trauma team activation
